# Esophageal Dysphagia in Children: State of the Art and Proposal for a Symptom-Based Diagnostic Approach

**DOI:** 10.3389/fped.2022.885308

**Published:** 2022-06-24

**Authors:** Gloria Lanzoni, Camilla Sembenini, Stefano Gastaldo, Letizia Leonardi, Vincenzo Pio Bentivoglio, Giovanna Faggian, Luca Bosa, Paola Gaio, Mara Cananzi

**Affiliations:** ^1^School of Specialty in Pediatrics, University Hospital of Padova, Padua, Italy; ^2^Unit of Pediatric Gastroenterology, Digestive Endoscopy, Hepatology and Care of the Child With Liver Transplantation, Department of Women’s and Children’s Health, University Hospital of Padova, Padua, Italy

**Keywords:** esophageal dysphagia, esophageal disorders, achalasia, eosinophilic esophagitis, peptic esophagitis, symptom based diagnosis, children, pediatric

## Abstract

Pediatric esophageal dysphagia (PED) is an infrequent condition that can be determined by a large number of disorders. The etiologic diagnosis is challenging due to overlapping clinical phenotypes and to the absence of pediatric diagnostic guidelines. This review aims to summarize the most relevant causes of ED during childhood, highlight the clinical scenarios of PED presentation and discuss the indications of available diagnostic tools. Available information supports that PED should always be investigated as it can underlie life-threatening conditions (e.g., foreign body ingestion, mediastinal tumors), represent the complication of benign disorders (e.g., peptic stenosis) or constitute the manifestation of organic diseases (e.g., eosinophilic esophagitis, achalasia). Therefore, the diagnosis of functional PED should be made only after excluding mucosal, structural, or motility esophageal abnormalities. Several clinical features may contribute to the diagnosis of PED. Among the latter, we identified several clinical key elements, relevant complementary-symptoms and predisposing factors, and organized them in a multi-level, hierarchical, circle diagram able to guide the clinician through the diagnostic work-up of PED. The most appropriate investigational method(s) should be chosen based on the diagnostic hypothesis: esophagogastroduodenoscopy has highest diagnostic yield for mucosal disorders, barium swallow has greater sensitivity in detecting achalasia and structural abnormalities, chest CT/MR inform on the mediastinum, manometry is most sensitive in detecting motility disorders, while pH-MII measures gastroesophageal reflux. Further studies are needed to define the epidemiology of PED, determine the prevalence of individual underlying etiologies, and assess the diagnostic value of investigational methods as to develop a reliable diagnostic algorithm.

## Introduction

**FIGURE 1:**
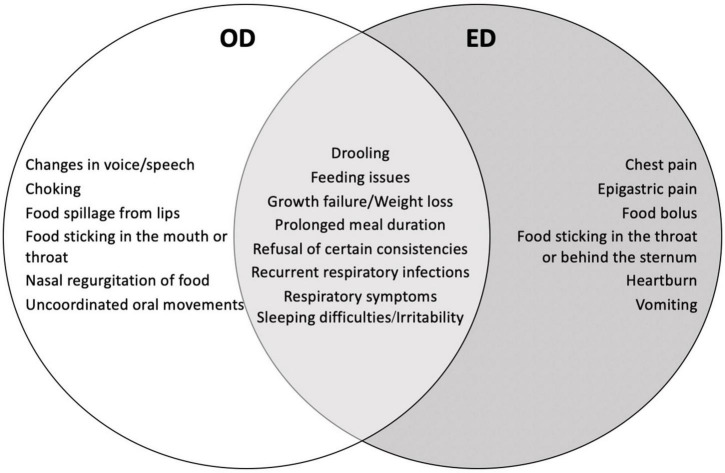
Common and peculiar clinical features of oropharyngeal and esophageal dysphagia (71, 72).

Dysphagia is defined as a disruption in the swallowing process that can involve any structure of the upper gastrointestinal (UGI) tract from the lips to the lower esophageal sphincter. The anatomical classification distinguishes oropharyngeal dysphagia (OD), in which the oral or pharyngeal phases of the swallowing process are involved, from esophageal dysphagia (ED) in which the underlying cause arises from the esophageal body or from the esophageal sphincters (1). Despite significant physiopathological differences, the distinction between OD and ED may be challenging, due to overlapping clinical presentation, especially in infants and young children (Figure 1). Regardless of the site involved, dysphagia can pose significant health issues and impair the child’s quality of life: the inability to eat properly can lead to dehydration and failure to thrive; the aspiration of food into the airways may cause recurrent respiratory infections and choking episodes; and, the difficulty in swallowing can elicit social isolation, anxiety, and depression (1–3).

Epidemiologic data regarding the prevalence of dysphagia in children are lacking. A nationwide US survey estimates that 0.9% of children, from 3 to 17 years of age, suffer from swallowing disorders with equal distribution between genders and mean age at onset of 8.2 years (4). While OD is prevalent in children with neurological impairment, the causes of dysphagia are more heterogeneous in children without developmental issues with ED being more relevant (5–7).

Pediatric ED (PED) can be the clinical manifestation of a wide variety of disorders. The majority of available studies focus on the specific etiologies of ED and, to the best of our knowledge, no guidelines or comprehensive recommendations are available for the diagnosis of PED. Due to the absence of a standardized diagnostic approach, the management of PED is extremely variable, especially as regards the employment of instrumental diagnostic techniques.

The aim of this review is to summarize the most relevant causes of PED, highlight the clinical key elements able to guide the diagnostic process, and discuss the available diagnostic tools with their specific indications. We also aim to propose a symptom-based diagnostic approach able to guide clinicians through the diagnostic work-up of PED.

## Materials and Methods

We performed a narrative review of the literature as to satisfy the Scale for the Assessment of Narrative Review Articles (SANRA) (8). The review was structured around three research questions, all relevant to the aim of the study: (i) which are the most relevant causes of PED; (ii) what clinical key elements are able to guide the diagnostic process; (iii) what are the available diagnostic tools. A search of the literature published after January 2000 was performed employing the Scopus, Embase, PubMed and Cochrane Library databases. The research was focused to papers written in English and to studies performed in human subjects aged from 0 to 18 years old. Multiple combinations of keywords were employed, referring to dysphagia as a symptom (e.g., “dysphagia”), to the pediatric age (e.g., “children”), to specific etiologies (e.g., “eosinophilic esophagitis”), or to diagnostic techniques (e.g., “esophageal manometry”). Papers not relevant to the topic were excluded by screening of titles and abstracts. Paper eligibility was confirmed by direct assessment of full-text articles. When available, practice guidelines, systematic reviews, meta-analysis, and RCTs, were considered the preferred references. Findings from selected studies were combined and summarized to provide the best available evidence to address the above-mentioned research questions.

Based on the revision of the literature, we developed a symptom-based diagnostic diagram aiming to guide the clinician toward the identification of the etiology underlying PED. To graphically represent this process, we coded in HTML-Javascript a dynamic sunburst chart. The diagram represents in a circular graph hierarchical relational data distributed on different levels. Each level represents the detalization of the previous in a centrifugal way.

## Results

### What Are the Most Relevant Causes of Pediatric Esophageal Dysphagia?

According to the underlying pathogenetic mechanism, esophageal disorders can be classified into mucosal, structural, and motility diseases as well as disorders caused by luminal esophageal obstruction (EO) or extrinsic compression (EC). The main causes of PED are reported in Table 1 and are synthetically described in Supplementary Table 1.

**TABLE 1:** Main causes of pediatric esophageal dysphagia along with representative disease features (peak age of incidence, predisposing factors, clinical features), and suggested diagnostic test(s).

	Disease	Peak age	Predisposing factors	Clinical features		Diagnostic test(s)
				Newborn- infant	Children-adolescents	
Mucosal disorders	GERD (25)	Adolescence	CI, EA, neurological impairment, previous surgery, SSc, RT.	Regurgitation, vomit, irritability, growth failure feeding or sleeping difficulties, anemia	Heartburn, epigastric pain, chest pain, sour burps, regurgitation, vomit,	EGD with biopsies, pH-MII
	Eosinophilic Esophagitis (24)	5–12 years	Atopy, EA	Feeding difficulties, vomit, growth failure	Food impaction, heartburn, vomit, chocking, gagging	EGD with biopsies
	Infectious esophagitis (27)	Any age	Immunodeficiency, prolonged steroid or antibiotic treatment	Odynophagia, chest pain, fever		EGD with biopsies and microbiological sampling
	Crohn’s disease (74)	Adolescence	–	Heartburn, chest pain, intestinal symptoms, fever, involuntary weight loss		EGD with biopsies
	Pill esophagitis (42)	8–18 years	Recent initiation of pill/tablet treatment	Odynophagia, chest pain		EGD with biopsies
	Caustic esophagitis (23)	<5 years, > 12 years	CI	Chest pain, sore throat, odynophagia, heartburn		EGD with biopsies
	Radiation esophagitis (75)	Any age	RT	Chest pain, sore throat, o dynophagia, heartburn		EGD with biopsies
Motility disorders	Achalasia (45)	7–12 years	Down’s and triple A syndromes, Pierre-Robin sequence	Paradoxycal ED, regurgitation of undigested food, feeding difficulties, chest pain, weight loss, respiratory symptoms		HREM, barium swallow, EGD
	Systemic sclerosis (40)	8–11 years	–		Food impaction, heartburn, regurgitation, chest pain, SSc extra-intestinal features	Barium swallow, EGD with biopsies, pH-MII, HREM
Structural disorders	Esophageal atresia (20)	Neonate	Concomitant malformations	Respiratory distress, impossible NG tube insertion, drooling	–	Chest X-ray, barium swallow
	Congenital stenosis (21)	Weaning time	–	Feeding difficulties, regurgitation of undigested food, respiratory symptoms-	Food impaction	Barium swallow, EGD
	Acquired stenosis/rings (35, 76)	Any age	CI, GERD, EoE, previous surgery, RT	Feeding difficulties, regurgitation of undigested food, respiratory symptoms-	Food impaction	Barium swallow, EGD
	Diverticula (77)	Any age	Motility disorders, previous surgery	Choking, respiratory symptoms, regurgitation of undigested food		Barium swallow, EGD
	Duplication cyst (78)	Any age	–	Respiratory symptoms		Barium swallow, EGD, chest CT scan or MRI
Luminal obstruction	Foreign body ingestion (15)	6 mths-3 years	–	Drooling, food refusal, retching, vomit, chest pain, stridor, cough		Chest X-ray, EGD
	Food impaction (15)	5–12 years	Achalasia, EoE, rings, stenoses, previous surgery	Drooling, feeding difficulties, retching, vomit, chest pain, stridor, cough		EGD with biopsies
Extrinsic compression	Vascular anomalies (14)	Any age	Congenital heart defects, Down’s syndrome	Respiratory symptoms		Barium swallow, Chest CT or MRI with angio
	Mediastinal disorders (33)	Any age	–	Respiratory symptoms, fever, weight loss		Chest X-ray, barium swallow, chest CT or MRI

From an epidemiological point of view, mucosal disorders constitute the most relevant cause of PED, with gastroesophageal reflux disease (GERD) and eosinophilic esophagitis (EoE) being the more frequent. The prevalence of GERD in children is 6% with an incidence that progressively increases with age (9). With a prevalence of 22.7 cases/100,000, EoE is far less frequent than GERD but is the leading cause of food impaction which constitutes the presenting symptom of disease in up to the 22% of children (6, 10). Primary esophageal motility disorders are a group of rare conditions subdivided, according to the Chicago Classification 4.0, into disorders of the esophagogastric junction outflow (i.e., Achalasia, Esophagogastric Junction Outflow Obstruction) and disorders of peristalsis [i.e., Distal Esophageal Spasm (DES), Hypercontractile Esophagus (nutcracker esophagus), Ineffective Esophageal Motility (IEM), and Absent Contractility] (11). Among the latter, achalasia is the one most commonly reported in the pediatric population but remains a rare disease with an incidence of 0.18 cases/100.000 children (12). No data are available regarding the epidemiology of the other primary esophageal motility disorders in children. Esophageal atresia (EA) has a prevalence of 0.7–3.2 cases/10,000 births and is the main representative of structural disorders causing PED (13). Dysphagia lusoria, due to an aberrant right subclavian artery, is the most frequent cause of esophageal EC (14). The esophagus is the most common site for an acute foreign body (FB) in the gastrointestinal tract. With a peak incidence between the ages of 6 months and 3 years, children make up the 80% of patients presenting to emergency departments with an esophageal FB (15).

When, after an appropriate evaluation, ED cannot be explained by another condition, functional dysphagia can be considered in the differential diagnosis. While it is the most prevalent esophageal disorder in adults, no data are available regarding its prevalence in the pediatric population (16). The diagnosis relies on the Rome IV criteria which define functional dysphagia as a sense of solid and/or liquid food sticking, lodging, or passing abnormally through the esophagus that arises in addition to all of the following criteria: (i) symptom onset at least 6 months before evaluation; (ii) symptom frequency of at least once a week; (iii) absence of evidence that esophageal mucosal or structural abnormality is the cause of the symptom; (iv) absence of evidence that gastroesophageal reflux or EoE is the cause of the symptom; (v) absence of major esophageal motor disorders (17).

### What Are the Clinical Key Diagnostic Elements for the Diagnosis of Pediatric Esophageal Dysphagia?

Children with ED may present with a variety of complaints ranging from a sensation of food getting stuck in the throat or behind the sternum to a proper inability to swallow any kind of food or beverage with associated drooling (18). In the case of long-standing ED, compensatory behaviors, such as chewing for prolonged periods of time, using water or other lubricants to facilitate the passage of food, or avoiding problematic ingredients, can accompany and somewhat mask the presence of true dysphagia and therefore must always be investigated (19).

Infants usually present with non-specific complaints such as feeding difficulties and bolus regurgitation, thus making it more challenging to recognize ED in this age group.

Our review of the literature highlighted the following as relevant clinical elements able to guide the diagnostic work-up of PED.

#### Age at Presentation

**FIGURE 2:**
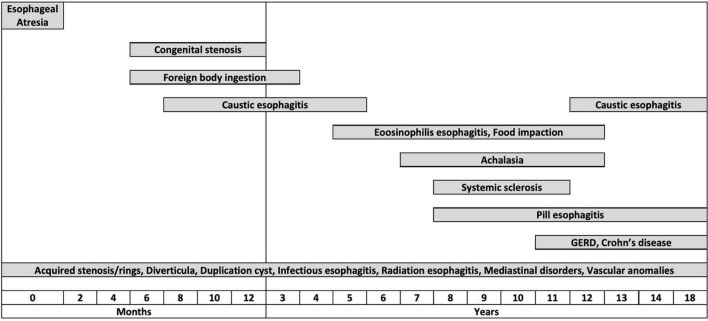
Graphical representation of the distribution of the different esophageal disorders by age groups.

While some disorders causing ED can develop at any age [e.g., infectious esophagitis (IE)], other conditions tend to arise at a typical age (Figure 2). Congenital disorders usually occur in neonates or infants: EA always presents within the first few hours of life (20), while congenital esophageal stenoses usually become evident at weaning or after the introduction of solid foods (21). FB ingestion (FBI) is typical of infants and preschool children (22). Caustic ingestion (CI) occurs in a bimodal fashion: accidental ingestion is most common under 5 years of age, while intentional ingestion, related to attempted suicide, mainly occurs in adolescents (23). EoE usually occurs in children between 5 and 12 years old (6, 24), and the incidence of GERD increases with age (9, 25).

#### Modality of Esophageal Dysphagia Onset

The majority of esophageal disorders presenting with dysphagia cause a chronic or intermittent dysphagia with or without a progressive pattern.

ED can also present acutely with a sudden inability to swallow solids and/or liquids. Under these circumstances, EO should always be suspected, especially if sialorrhea is present. While FBI mostly concerns infants and toddlers with no underlying esophageal disfunction, food impaction is the most common cause of acute EO in older children and teenagers. The latter is usually the presenting symptom of an esophageal disorder, among which EoE is the most frequent (6, 26). Less frequent causes consist of peptic-, caustic- or radiation-induced strictures, Schatzki’s rings, esophageal diverticula, achalasia, and post-surgical complications (15). IE also presents acutely but, in this case, obstructive symptoms are usually absent, while fever and pain are predominant (27).

#### Food Consistency

ED can be categorized based on the consistency of foods for which the swallowing process is impaired. Orthodox dysphagia is characterized by an impairment in managing solid foods which may later involve liquids, while paradoxical dysphagia presents with a difficulty in managing liquids from symptom onset. The first is a consequence of a mechanical obstruction of the esophagus (either intrinsic or extrinsic), while the latter is secondary to motility disorders (28, 29).

#### Associated Symptoms

ED may be an isolated symptom or occur in association with other clinical findings.

UGI symptoms such as heartburn, epigastric pain, chest pain, and regurgitation are usually reported in GERD (7, 25). Food bolus impaction points toward the diagnosis of EoE (24). The combination of bolus regurgitation, chest pain, weight loss, and paradoxical dysphagia constitute the typical presentation of achalasia (30, 31).

Respiratory symptoms such as nocturnal cough and recurrent respiratory infections are also frequently reported in patients with esophageal achalasia due to recurrent aspiration or tracheal compression from the dilated esophagus (32). Sore throat, hoarseness, nocturnal cough, wheezing, and recurrent respiratory infections have been identified as atypical symptoms of GERD (25). Moreover, respiratory distress, stridor and wheezing can accompany dysphagia in the presence of any chest mass or vascular malformation causing a compression on mediastinal structures (14, 33).

Fever in association to odynophagia and chest pain suggest the diagnosis of IE or, in case of a compatible clinical history, may prompt the possibility of FBI complicated with esophageal perforation and acute mediastinitis (34). Mediastinal masses can be associated to the presence of paraneoplastic fever (33).

#### Comorbidities

Several pre-existing conditions may predispose to the onset of dysphagia. Former esophageal surgery, radiation therapy and CI can lead to acquired stenosis and predispose to GERD (35). With a reported incidence as high as 70%, GERD is also common in children with neurological impairment (5). Achalasia has been linked to Down’s syndrome (36), Pierre-Robin sequence and other rare genetic disorders (37). Congenital heart defects and Down’s Syndrome are associated to vascular anomalies causing EC (14). Allergologic disorders and EA constitute well-established risk factors for the development of EoE (24, 38). Primary or acquired immunodeficiencies as well as prolonged antibiotic and steroid treatment favor the development of IE (34, 39). Immune-mediated disorders, such as systemic sclerosis (40) and Crohn’s disease can rarely involve the esophagus causing ED (41). Ongoing treatment with tablets/capsules should raise the possibility of pill-induced esophagitis especially when there is a close temporal relation (<10 days) with the use of NSAIDs, antibiotics (e.g., doxycycline, amoxicillin), L-arginine and iron, and the drug has been taken with little water at bedtime (42, 43).

### What Are the Available Diagnostic Tools?

The diagnostic work-up of PED can include a luminal, mucosal, anatomic, and functional assessment of the esophagus. Several diagnostic methodologies are available, the specific indications and diagnostic rates of which are discussed below. Moreover, pros and cons of the main diagnostic tools are summarized in Table 2.

**TABLE 2:** Pros and cons of the main diagnostic tools employed in the assessment of PED (48, 73).

	Pros	Cons
Esophagogastroduodenoscopy (EGD)	• Enables direct visualization of the esophageal lumen and mucosa • Allow histological and microbiological sampling • Allows endoscopic removal of FB • Allows pneumatic dilation of the esophagus	• Sedation needed • Unable to diagnose motility disorders • Rare complications (e.g., infection, perforation, bleeding)
pH-multichannel intraluminal impedance (pH-MII)	• Allows precise evaluation of GER • Can be coupled with EGD	• Requires patient cooperation • No anatomical or motility information
Barium swallow	• Widely available • Provides good anatomical detail of the esophagus • Can identify motility disorders (e.g., achalasia) • Can demonstrate an extrinsic compression of the esophagus	• Radiation exposure • Requires patient cooperation • Cannot be performed in patients with bowel obstruction or suspected perforation • Aspiration of barium can lead to hypersensitivity pneumonitis, and barium leakage through perforation can lead to mediastinitis and peritonitis
High-resolution esophageal manometry (HREM)	• Allows characterization of motility disorders • Can be used to classify esophageal achalasia subtypes, define treatment and prognosis • May be used to characterize congenital stenoses	• Requires patient cooperation • No direct visualization of the esophageal anatomy nor mucosa
Chest X-ray	• Can identify radio-opaque foreign bodies • Can identify mediastinal enlargement and pneumomediastinum	• Radiation exposure
Chest CT/MRI	• Allow identification of structural disorders or extrinsic esophageal compression • Coupled with angiography represent the gold standard for the diagnosis of vascular anomalies causing dysphagia.	• Radiation exposure (only for CT) • Sedation needed in young children

#### Esophagogastroduodenoscopy

The methods and the indications for performing EGD in children with ED have been reported by the ESPGHAN-ESGE guidelines (23).

In the context of GERD-related dysphagia, EGD is useful for: (i) defining the obstructive vs. non-obstructive nature of the symptom (stenosis vs. dysmotility); (ii) detect conditions that predispose to GERD (e.g., hiatal hernia); (iii) exclude conditions mimicking GERD such as EoE (7, 23). Indeed, the detection of > 15 eosinophils per HPF in at least 1 biopsy specimen of the esophageal mucosa is the gold standard for the diagnosis of EoE and the performance of 1–2 biopsies from the proximal, middle, and distal esophagus provides for a 97–100% sensitivity in the detection of esophageal eosinophilia (23, 24, 44). In IE, EGD reveals typical mucosal changes and provide for a microbiological diagnosis (39). EGD is the method of choice for detecting the characteristic features of pill-induced esophagitis which consist of single or multiple ulcers, surrounded by normal mucosa, usually located in the middle-third of the esophagus (42, 43).

EGD plays a diagnostic and therapeutic role in acute EO. Any symptomatic patient with suspected FBI should emergently undergo EGD as well as asymptomatic patients who swallowed sharp FB or button butteries. The endoscopic removal of asymptomatic blunt esophageal FB is also recommended within 24 h from the ingestion (23).

EGD can rise the suspicion of achalasia, and exclude pseudoachalasia, but is not diagnostic *per se* and may miss early disease. Abnormalities are present in the 90% of affected children with most common findings consisting of residual food in the esophagus (75%), closed stomach cardia (73%), esophageal enlargement (58%), and mucosal lesions (28%) (45). In patients with symptom relapse after surgery, EGD is useful to assess for reflux vs. recurrence of achalasia (30, 31).

EGD may also reveal a narrowing of the esophageal lumen caused by structural lesions or EC. In these circumstances, however, the definite diagnosis usually requires the employment of other diagnostic modalities including CT, MRI, and endoscopic ultrasound (46).

In children with a truly negative endoscopy (i.e., after an adequate biopsy sampling), radiologic studies and esophageal function tests should be warranted to investigate for subtle structural lesions (e.g., rings), EC and motility disorders (47).

#### Radiologic Techniques

Plain chest X-ray has a limited role in the assessment of PED. It can identify radiopaque FBs stuck into the esophagus, exclude the presence of pulmonary issues when fever or respiratory symptoms are present, and screen for pneumomediastinum or gross mediastinal enlargements (22).

Chest CT and MRI are needed to investigate the mediastinum when an esophageal EC is suspected. When coupled with angiography, these methodologies are the gold standard for the diagnosis of vascular anomalies causing dysphagia (14).

Barium swallow (BS) is useful in diagnosing esophageal webs, rings, stenoses, diverticula, tumors and EC (48, 49). Virtually all children with achalasia present at least one typical BS feature; the most common consist of slow contrast transit through the esophagus into the stomach (96.1%), esophageal dilation (94.1%), “bird’s beak” sign (82.4%), contrast retention in the esophagus (74%), and peristaltic anomalies (62%) (31, 45, 50). Besides providing excellent mucosal detail, BS is seldom used for the diagnosis of esophagitis and cannot establish or negate a diagnosis of GERD. However, it can detect conditions predisposing to GERD (e.g., hiatal hernia) and differentiate an obstructing vs. a slipped/loose fundoplication (7).

#### Esophageal Function Tests

pH-metry or dual pH-multichannel intraluminal impedance (pH-MII) are not routinely recommended for the diagnosis of GERD (7). However, they can be helpful in the context of GERD-related dysphagia, as to: (i) correlate the symptom with acid/non-acid reflux events; (ii) clarify the role of acid/non-acid reflux in the etiology of esophagitis; (iii) evaluate for reflux in patients with EoE as these two conditions are not mutually exclusive; (iv) determine the efficacy of acid suppression in case of persisting dysphagia during treatment; (v) assess the onset of GERD after surgical and endoscopic treatments for achalasia (7, 24, 51). When available, pH-MII should be preferred to pH-monitoring and considered the gold standard diagnostic technique for the detection of GERD in the pediatric population (52). Indeed, since it allows the measurement of all reflux events irrespective of pH, pH-MII allows the detection of non-acid reflux episodes which have a high prevalence in the pediatric population, can be carried out on or off reflux therapy, and may be performed during continuous enteral feeding (49, 50, 52).

High-resolution esophageal manometry (HREM) represents the gold standard for the diagnosis of esophageal motility disorders, particularly achalasia (30, 31, 53). This technique uses a standardized swallow challenge to measure peristaltic pressures throughout the esophageal body (54). The Chicago classification algorithm classifies esophageal motility disorders according to manometric measurements of esophageal peristalsis (DCI, or Distal Contractile Integral, is the amplitude x duration x length [mmHg*s*cm] of the distal esophageal contraction above 20 mmHg, from the transition zone to the proximal margin of the lower esophageal sphincter; DL, or Distal Latency, is the interval between upper esophageal sphincter relaxation and the distal contraction) and esophago-gastric junction relaxation (IRP4s, or Integrated Relaxation Pressure, measured as the mean of the 4 s of lowest pressure across the lower esophageal sphincter in the 10 s window after swallow) (11). HREM has been recently combined with intraluminal impedance mapping (HRIM, High Resolution Impedance Manometry). This approach allows to directly evaluate the transit of the esophageal bolus using pressure-impedance metrics (IBP, or Intra-bolus Distension Pressure, indicating flow resistance; BFT, or Bolus Flow Time, indicating trans-esophagogastric junction emptying; and Impedance Ratio, indicating bolus transit failure), thus increasing the yield for detecting esophageal motility disorders (54). The use of the above-mentioned techniques has been validated in children (55), although some distinctions should be considered when interpreting the results. Patient characteristics and esophageal length and caliber have a significant impact on esophageal pressure topography metrics, thus, in pediatric patients reference ranges should be adjusted for size (55). Moreover, children under 7 years of age are often unable to tolerate the procedure and patient movement and crying can produce measurement artifacts that can impair study interpretation (54).

#### Questionnaires and Clinical Scales

As said above (section “What Are the Clinical Key Diagnostic Elements for the Diagnosis of Pediatric Esophageal Dysphagia”, the clinical identification as well as the measurement of PED pose several challenges. To address this issue, questionnaires and clinical outcome metrics (COMs) have been developed to identify, quantify, and monitor the presence of a swallowing problem either independently from the underlying etiology or in specific pathological contexts (19).

The Eating Assessment Tool (EAT-10) (56) and its pediatric modified version (57) (PEDI-EAT-10) are symptom-based questionnaires that support the clinician in identifying the presence of a swallowing problem and in assessing the risk of penetration/aspiration (Supplementary Tables 2, 3).

The Reflux Symptom Index (RSI) (58) is a self-administered nine-item instrument to detect laryngopharyngeal reflux (Supplementary Table 4). It has been recently proven to be inaccurate in assessing the presence of gastroesophageal reflux in children and infants when compared to pH-MII. Indeed, although the number of acid refluxes directly correlates with the RSI, a normal RSI value does not exclude the presence of a pathological MII-pH (59).

Several scoring systems have been developed for the clinical evaluation of EoE. In adults, the Eosinophilic Esophagitis Activity Index was found to have the best validity and responsiveness in a systematic review: this tool focuses on dysphagia induced by food of different consistencies and on behavioral adaptation in daily life (60, 61). In children, the Pediatric Eosinophilic Esophagitis Symptom Score (PEESS) (62) has been validated to assess dysphagia in subjects from 2 to 18 years of age based on child and/or parent proxy scores (Supplementary Tables 5, 6). The aforementioned indexes are useful in assessing and monitoring EoE symptoms; nevertheless, they cannot be used to make assumptions on the biological activity of EoE, as they have a predictive unsatisfactory value (63).

The Eckardt score has been specifically developed for esophageal achalasia to establish clinical disease severity at diagnosis and to evaluate treatment efficacy during follow-up (30, 64, 65). The score is based on the three main symptoms associated with achalasia (dysphagia, regurgitation, and chest pain) in adjunct to weight loss included as a marker of the patient’s capacity to maintain nutrition (Supplementary Table 7). Unfortunately, the application of the Eckardt score in children is limited since the questionnaire is only validated for subjects older than 14 years of age (31, 65). A disease-specific quality of life measure has been developed and validated for children with achalasia (66). However, despite the availability of this questionnaire since 2010, it is seldom employed in pediatric clinical practice (31).

## Discussion

Our review of the literature shows that PED is caused by a heterogeneous group of disorders, among which EoE and GERD are the most frequent, summarizes the diverse clinical scenarios of PED presentation, and highlights the most appropriate investigational method(s) based on the diagnostic hypothesis. Despite available knowledge regarding individual etiologies, this revision also confirms the lack of pediatric epidemiological data and the absence of diagnostic guidelines regarding PED as a symptom.

In adults, ED is considered an alarm sign that may underlie the presence of esophageal carcinoma (67, 68). In children, ED is not clearly recognized as a red flag given the rarity of esophageal cancer in this age group. However, available information regarding the etiology of PED supports that this symptom should always be investigated as it can underlie life-threatening conditions (e.g., FBI, CI, mediastinal tumors), represent the complication of benign esophageal disorders (e.g., GERD-related stenosis) or constitute the clinical manifestation of an organic disease (e.g., EoE, achalasia). Therefore, in children as in adults, the diagnosis of functional dysphagia should only be made after demonstrating the absence of mucosal, structural, or esophageal motility abnormalities (17).

Several diagnostic protocols are available for the diagnosis of ED but none has been specifically made for children. No sufficient data are available to develop a diagnostic algorithm for PED or advocate for the superiority of a diagnostic test over another as first-line investigation.

**FIGURE 3:**
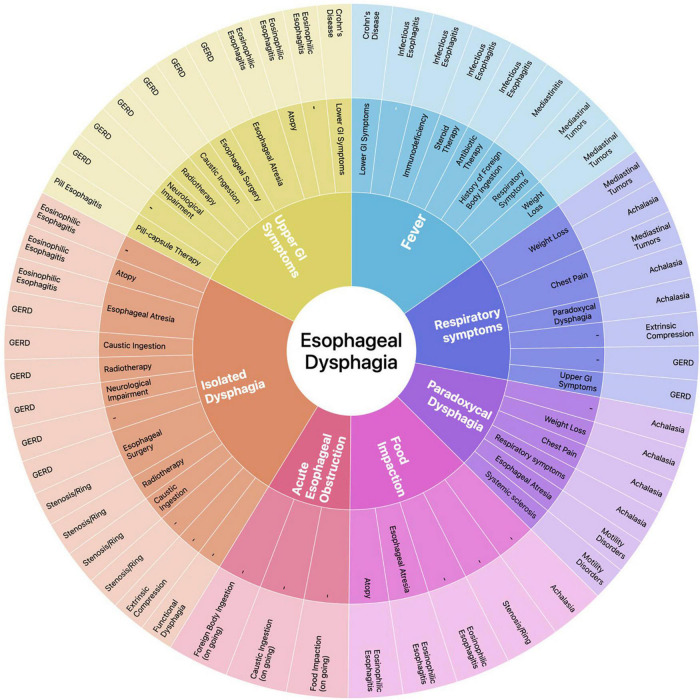
Sunburst diagram representing our proposed symptom-based diagnostic approach to ED. The diagram consists of four concentric circles (or levels) which, from the inside to the outside, represent in a hierarchical relation: (1) the clinical key elements that guide the diagnostic process; (2) relevant complementary symptoms and disease factors predisposing to a specific etiology that, in addition to the corresponding clinical key element, may address the differential diagnosis; (3) the most probable etiologic diagnosis/diagnoses of ED based on the clinical elements selected in the underlying levels. Each clinical key element is represented in a different color and this color is maintained throughout the corresponding section of the graph, albeit with different tones along the different levels (i.e., rings 1, 2, 3). Relevant complementary symptoms and predisposing factors in ring 2 make the etiologic diagnosis in ring 3 more likely; when no element is indicated it means that the final diagnosis may not be associated to any other clinical element. A dynamic version of the diagram is available in Supplementary Material.

We propose a diagnostic approach tailored on the main diagnostic suspect(s) deriving from the clinical evaluation of the child. To this end, we have identified six clinical key elements capable of recognizing patients who require an immediate medical evaluation (i.e., signs of acute EO and fever) or directing toward a diagnosis (i.e., history of food impaction, isolated dysphagia, paradoxical dysphagia, respiratory symptoms, UGI symptoms). As none of abovementioned clinical key elements can be univocally associated to a single etiology, we have also identified relevant complementary symptoms and predisposing factors that, combined to clinical key elements, can support the clinician throughout the diagnostic process. This symptom-based approach is graphically represented in a circular graph with hierarchical relational data distributed on different levels (Figure 3). Once one or more diagnostic hypotheses have been made, the most appropriate investigational method(s) should be chosen based on the specificities of individual tests. EGD has highest diagnostic yield for the evaluation of luminal and mucosal disorders. BS has a greater sensitivity in detecting achalasia and structural abnormalities, is non-invasive and can be used to confirm indication and define setting (diagnostic vs. operative) prior to perform EGD (28, 69, 70). Chest CT and MR provide detailed anatomical information on the mediastinum. Manometry is the most sensitive technique to detect motility disorders, while pH-MII evaluates the presence of acid/non-acid reflux (48, 49, 70).

To the best of our knowledge, this is the first comprehensive review regarding PED. Large scale studies are needed to define the epidemiology of PED, determine the prevalence of underlying etiologies, and assess the diagnostic value of available investigational methods as to establish the relative risk of disease and develop a reliable diagnostic algorithm. Prospective studies should be conducted, or a machine-learning approach implemented, to validate our symptom-based approach for the etiological definition of PED.

## Author Contributions

GL, PG, and MC wrote the manuscript, prepared tables and figures which all authors reviewed. GL, CS, SG, LL, VPB, and PG performed the literature search and summarized the results. GF and LB performed a critical review of the findings and participated in the preparation of the manuscript. All authors contributed to the article and approved the submitted version.

## Conflict of Interest

The authors declare that the research was conducted in the absence of any commercial or financial relationships that could be construed as a potential conflict of interest.

## Publisher’s Note

All claims expressed in this article are solely those of the authors and do not necessarily represent those of their affiliated organizations, or those of the publisher, the editors and the reviewers. Any product that may be evaluated in this article, or claim that may be made by its manufacturer, is not guaranteed or endorsed by the publisher.

## Glossary

Antibiotic therapy, recent or ongoing prolonged antibiotic therapy; Atopy, former diagnosis of one or more atopic disorders; CI, history of caustic ingestion; Esophageal atresia, previous surgery for esophageal atresia; GERD, gastroesophageal reflux disease; Immunodeficiency, former diagnosis of congenital or acquired immunodeficiency; Lower GI symptoms, lower gastrointestinal symptoms (e.g., abdominal pain, chronic diarrhea, blood in stool); Neurologic impairment, child with neurological disability; Pill-capsule therapy, recent or ongoing treatment with pill or capsules; Radiotherapy, previous radiation therapy; Respiratory symptoms, associated respiratory symptoms (e.g., cough, recurrent infections); Steroid therapy, recent or ongoing prolonged steroid treatment; Esophageal surgery, previous surgery on the esophagus; Systemic sclerosis, former diagnosis of systemic sclerosis; Upper GI symptoms, upper gastrointestinal symptoms (e.g., heartburn, epigastric pain, sour burps, regurgitation, vomit).

## Abbreviations

BS: Barium swallow
CI: caustic ingestion
CT: computed tomography
EA: esophageal atresia
EC: extrinsic compression
ED: Esophageal dysphagia
EGD: esophagogastroduodenoscopy
EoE: eosinophilic esophagitis
EO: esophageal obstruction
FB: foreign body
FBI: foreign body ingestion
GERD: gastroesophageal reflux disease
IE: infectious esophagitis
OD: oropharyngeal dysphagia
MRI: magnetic resonance
PED: pediatric esophageal dysphagia
UGI: upper gastrointestinal.
