# Supplementary material for: Esophageal Dysphagia in Children: State of the Art and Proposal for a Symptom-Based Diagnostic Approach
Source: Front Pediatr. 2022 Jun 24;10:885308. doi: 10.3389/fped.2022.885308 (PMC9263077; doi:10.3389/fped.2022.885308)
Supplement: Supplementary file 1 [file Data_Sheet_1.pdf]

SUPPLEMENTARY MATERIAL

CAUSES OF PEDIATRIC ESOPHAGEAL DYSPHAGIA

According to the underlying pathogenetic mechanism, esophageal disorders can be classified into mucosal, structural and motility diseases as well as disorders caused by luminal obstruction or extrinsic compression of the esophagus. When none of the above etiologies can be recognized, functional dysphagia can also be included in the differential diagnosis (**supplementary Table 1**).

The main causes of pediatric ED are synthetically described below. The description of foreign body and caustic ingestion as well as of mediastinal disorders goes beyond the aim of this review, thus these topics will not be treated in the next paragraphs.

Supplementary table 1. Classification of esophageal disorders causing dysphagia in children.

**Mucosal disorders**	**Motility disorders**	**Structural disorders**	**Luminal obstruction**	**Extrinsic compression**	**Functional disorders**
GERD and peptic esophagitis	Achalasia	Esophageal atresia	Foreign body ingestion	Congenital vascular anomalies	Functional dysphagia
Eosinophilic esophagitis	Esophagogastric Junction Outflow Obstruction	Esophageal stenosis	Food impaction	Mediastinal tumors	
Infectious esophagitis	Distal esophageal spasm	Esophageal rings		Mediastinal lympha-denopathies	
Crohn's disease	Hypercontractile esophagus	Esophageal diverticula			
Pill esophagtis	Ineffective esophageal motility and absent contractility	Esophageal duplication cyst			
Caustic ingestion	Systemic sclerosis				
Radiation esophagitis					

1. Mucosal disorders

1.1 Reflux disease (GERD) and peptic esophagitis

Gastroesophageal reflux disease (GERD) is defined as the presence of symptoms and/or complications due to the passage of gastric content into the esophagus (1). A cross-sectional observational study showed a prevalence equal to 6.2% in patients between 0 and 17 years of age (2).

In infants and young children, GERD usually presents with growth failure, regurgitation/vomiting, irritability, feeding or sleeping difficulties, anemia. In children and adolescents, heartburn, epigastric pain, chest pain, dysphagia, nocturnal pain, and sour burps are more frequent. GERD can also present with extra esophageal, or atypical symptoms, such as cough, wheezing, recurrent pneumonia, sore throat, hoarseness, chronic sinusitis, laryngitis or dental erosion (3). Dysphagia has been reported as a symptom associated with GERD usually related to the presence of erosive esophagitis or peptic esophageal stenosis (1, 4). Esophago-gastro-duodenoscopy (EGD) with biopsies and pH monitoring studies are used to confirm the diagnosis. Moreover, histology is useful to differentiate other causes of esophagitis (1).

1.2 Eosinophilic Esophagitis (EoE)

EoE is a chronic immune-mediated inflammatory disease. A recent metanalysis reported an incidence of 5.1 cases/100.000 per year and a prevalence equal to 22.7 cases/100000 in children (5). Epidemiologic data show that EoE is more frequent in males (O.R. male vs female 2.01), with a peak onset between 5 to 12 years of age, and is often associated with atopic diseases (5-7). Isolated ED frequently represents the presenting symptom of disease especially in adolescents. Food bolus impaction constitutes the first manifestation of EoE in up to the 21,7% of cases (6). Other features such as feeding difficulties, recurrent vomiting and GERD-like symptoms have also been described, particularly in younger children (7). EGD with esophageal biopsies represents the gold standard for the diagnosis of EoE. To maximize diagnostic sensitivity, it is recommended to perform at least 1 or 2 biopsies from a minimum of 3 esophageal sites regardless of the endoscopic appearance of the esophagus (8). The presence of 15 eosinophils per high-power field in at least 1 histologic sample represents the minimum diagnostic threshold to confirm EoE (7, 9).

1.3 Infectious esophagitis

Infectious esophagitis (IE) represents worldwide the third leading cause of esophagitis after GERD and EoE. IE can be caused by bacterial, viral, fungal, and parasitic infections. The most common agent is Candida albicans, followed by HSV and CMV (10). History of primary or acquired immunodeficiency, antibiotic therapy, prolonged hospitalization, and malnutrition represent the main risk factors for the development of IE at all ages (10, 11). The presenting symptoms mainly consist of dysphagia, odynophagia, heartburn, hematemesis, and fever (10, 11). To confirm diagnosis and define etiology, EGD with biopsies and collection of microbiological samples are required (10, 12).

1.4 Crohn's Disease

Crohn's disease is a systemic immune-mediated inflammatory condition which can involve any site of the gastrointestinal tract. The reported prevalence of CD's esophageal involvement in pediatric patients is highly variable ranging between 6.5% to 42% (13, 14). Symptoms specifically related to esophageal involvement, such as heartburn, chest pain and dysphagia have been reported in 14% of cases (14). Sporadic reports of esophageal Crohn's disease presenting with symptomatic esophageal stricture have also been described (15). EGD with biopsies allows the identification of esophageal involvement (14).

1.5 Pill esophagitis

Pill-esophagitis has been seldom reported during the pediatric age especially in school-aged children and adolescents who are able to swallow pills or tablets. The most frequently reported symptoms consist of dysphagia, retrosternal pain, and odynophagia.

Ongoing treatment with tablets or capsules, should raise the possibility of pill-induced esophagitis especially when there is a close temporal relation (< 10 days) with the use of non-steroidal anti-inflammatory drugs, antibiotics (*e.g.*, doxycycline, amoxicillin, clindamycin), L-arginine and ferrous sulfate and the drug has been taken with little water at bed time (16, 17).

EGD is the method of choice for detecting the characteristic features of pill-induced esophagitis which consist of single or multiple (usually kissing) ulcers, surrounded by normal mucosa, usually located in the middle-third of the esophagus (16, 17).

1.6 Radiation esophagitis

Radiation esophagitis is an inflammation of the esophagus due to radiation. Radiation esophagitis is an adverse effect that develops in individuals receiving radiation cancer therapy, most commonly

for breast, lung, and other lymphomas. It occurs very rarely in the pediatric age. Certain risk factors may predispose patients to a high risk of developing radiation esophagitis. These include white race, older age, female sex, poor initial performance status, low body mass index, and gastroesophageal reflux disease. Symptoms present two to three weeks after the initial therapy and include throat pain, dysphagia, and the sensation that food is stuck. Although radiation esophagitis is relatively benign and self-limiting, in severe cases, it can cause ulcerations, esophageal perforations, tracheoesophageal fistula and stricture development (18).

2. Motility disorders

The Chicago Classification categorizes primary esophageal motility disorders via an algorithmic scheme using metrics from esophageal high-resolution manometry (HRM) with esophageal pressure topography (EPT) into disorders of esophagogastric junction outflow (*i.e.*, Achalasia, Esophagogastric Junction Outflow Obstruction) and disorders of peristalsis (*i.e.*, Distal Esophageal Spasm [DES], Hypercontractile Esophagus [nutcracker esophagus], Ineffective Esophageal Motility [IEM], and Absent Contractility) (19).
Systemic sclerosis is an acquired disorder of esophageal motility.

2.1 Disorders of esophagogastric junction (EGJ) outflow

2.1.1 Achalasia

Achalasia is an esophageal motility disorder characterized by impaired relaxation of the lower esophageal sphincter (LES) and abnormal peristalsis of the esophageal body. The underlying etiology is not yet completely elucidated but it implicates the loss of ganglionic cells in the distal esophagus causing an imbalance between inhibitory and excitatory neurotransmitters that ultimately results in dysmotility and LES dysfunction (20). With an incidence ranging between 0.18/100.000 children, achalasia is a rare pediatric disorder (21). Down Syndrome, Pierre-Robin sequence, Allgrove (Triple A) syndrome (OMIM #231550) and other rare genetic disorders (Achalasia-Microcephaly Syndrome [OMIM #200450], Alacrima-Achalasia-Mental Retardation Syndrome [OMIM #615510], Moyamoya disease 6 [OMIM #615750], familial dysautonomia, familial glucocorticoid insufficiency) have been associated to esophageal achalasia in children (22). Achalasia has usually an insidious onset with a mean age at diagnosis in children ranging between 7 and 12 years of age. Symptoms reported in

children and adolescents consist of progressive dysphagia, regurgitation/vomiting of undigested food, feeding problems, weight loss and chest pain. Nocturnal cough and recurrent respiratory infections are also been frequently described (23, 24). The diagnostic work-up includes endoscopy, barium upper GI series, and esophageal manometry (HREM) (25). Esophageal manometry allows the identification of the 3 subtypes of achalasia defined by the Chicago Classification which harbors relevant therapeutic and prognostic implications (20).

2.1.2 Esophagogastric Junction Outflow Obstruction (EGJOO)

EGJOO can be classified as a disorder of the esophagogastric junction motility when Type III achalasia diagnostic criteria are not fulfilled. The clinical presentation mainly consists of esophageal dysphagia (usually involving solid foods) and/or non-cardiac chest pain. Definitive diagnosis of clinically significant EGJOO requires evidence of obstruction by timed barium esophagogram (TBE). It is crucial to distinguish between clinically relevant EGJOO, that may represent an underlying pathologic motor disorder responsive to treatment, versus a clinically irrelevant manometric observation (19).

2.2 Disorders of peristalsis

Disorders of peristalsis should be considered when EGJ outflow disorders have been ruled out) (19).

2.2.1 Distal esophageal spasm (DES)

DES is a specific abnormal esophageal motor pattern characterized by spastic or premature contractions in the distal esophagus. Diagnosis of DES requires the presence of both clinically relevant symptoms (dysphagia and non-cardiac chest pain) and a conclusive manometric diagnosis of DES consisting of at least 20% premature contractions (19).

2.2.2 Hypercontractile esophagus (nutcracker esophagus)

Hypercontractile esophagus is the result of an excessive cholinergic drive with temporary asynchrony of circular and longitudinal muscle contraction (26). The clinical presentation is characterized by the presence of dysphagia and/or non-cardiac chest pain. Esophageal manometry (HREM) represents the main diagnostic tool. To confirm diagnosis Achalasia and DES should be excluded, as any other cause of mechanical obstruction (19).

2.2.3 Ineffective esophageal motility (IEM) and Absent contractility

IEM is defined as the presence of more than 70% ineffective swallows or at least 50% failed peristalsis at HREM. Absent contractility is characterized by the presence of 100% failed peristalsis. Both these conditions do not represent a specific diagnosis and can be the clinical manifestation of several disorders, mainly including neuropathies and myopathies. Supportive testing such as timed barium swallow can be used to demonstrate poor bolus transit and confirm diagnosis (19, 26).

2.3 Systemic Sclerosis

Systemic sclerosis (SSc) is a rare multisystemic disease with onset during childhood in about 10% of cases (mean age at onset 8-11 years with female predominance) (27). Gastrointestinal symptoms occur in 42% to 74% of cases with esophageal involvement being the most common feature (27-29). SSc causes fibrosis of the esophageal wall which leads to esophageal dysmotility. Esophageal dysmotility can lead to dysphagia per se but can also determine GERD with consequent ED (30). All patients with SSc should be evaluated for gastrointestinal symptoms. Symptomatic patients can be investigated with esophageal manometry, barium swallow and EGD to highlight the presence of esophageal and upper GI dysmotility and investigate for GERD, respectively (27, 30).

3. Structural disorders

3.1 Esophageal atresia

Esophageal atresia (EA) is the most common congenital abnormality of the esophagus. Available literature reports a prevalence ranging between 0.7 e 3.2 cases/10.000 births (31).

The cause of esophageal atresia is unknown but the association to other additional birth defects (*e.g.*, VACTERL and CHARGE syndrome) in half of affected children support the hypothesis that it consists of a malformative syndrome. Risk factors include older paternal age and assisted reproductive technology.

There are four types of esophageal atresia (www.cdc.gov):

- **Type A** is when the upper and lower parts of the esophagus do not connect and have closed ends. In this type, no parts of the esophagus attach to the trachea.
- **Type B** is very rare. In this type, the upper part of the esophagus is attached to the trachea, but the lower part of the esophagus has a closed end.

- **Type C** is the most common type. In this type, the upper part of the esophagus has a closed end, and the lower part of the esophagus is attached to the trachea, as is shown in the drawing.
- **Type D** is the rarest and most severe. In this type the upper and lower parts of the esophagus are not connected to each other, but each is connected separately to the trachea.

Diagnosis can be suspected in uterus, due to the presence of an absent/small stomach with polyhydramnios and confirmed soon after birth. Newborns usually present with respiratory distress. The impossible progression of a nasogastric tube and typical thoraco-abdominal X ray findings confirm the diagnosis.

Surgical repair is required in the first few days of life (32). Beyond neonatal age, children with surgically treated EA are prone to develop motility esophageal disorders and gastroesophageal reflux disease which can lead to ED (33).

3.2 Esophageal stenosis

Congenital esophageal stenosis (CES) is a rare anomaly resulting from incomplete separation of the respiratory tract from the primitive foregut. The incidence is estimated at 1 per 25.000 to 50.000 live births, with a slightly male predominance (34). An association with other anomalies, particularly esophageal atresia, is reported in 17–33% of the cases (35).

Three anatomic types of CES are distinguished: esophageal membranes (EM) or web, fibromuscular stenosis (FMS) and ectopic tracheobronchial remnants (TBR) (36). The last is the most common form, involving preferentially the lower third of the esophagus, while the other two are more commonly seen in the middle third.

The symptoms vary depending on the location and severity of the stenosis. Generally, high lesions present with respiratory symptoms, while low lesions present with vomiting. Usually, symptoms start around the weaning period with dysphagia to solids. However, some patients may become used to live with minor degrees of stenotic lesions, eventually seeking medical attention only as young adults due to food impaction, despite a life-long history of dysphagia (37).

The diagnostic gold standard consists of barium swallow (38). Esophagogram generally reveals a concentric, aperistaltic, sometimes asymmetric, narrowing of the upper, mid, or distal (frequently within 3.5cm of the gastric cardia) esophagus, with variable length (about 1–2cm), smooth contours and tapered borders. It is usually associated with proximal dilatation and GER can also be found (34). Endoscopy identifies stenosis, rules out esophagitis and foreign bodies and allows biopsy, when possible. More recently, endoscopic ultrasonography has also been advocated as a helpful

tool for CES diagnosis and pre-treatment assessment since it may distinguish TBR with cartilage from FMS (39). For greater diagnosis accuracy and pre-surgery study, magnetic resonance imaging (MRI) can also be performed, being preferred to CT scan given the young age group of most of the patients. When MRI is not available, CT scan with oral contrast is also an option. On MRI, congenital esophageal stenosis due to TBR will appear as an asymmetric wall thickening in the upper- mid- or distal esophagus, possibly with some hypointense areas on the T2-weighted sequences corresponding to the cartilage islands. On CT scan, wall thickening might also be depicted. Manometry may also be indicated to characterize congenital esophageal stenoses such as those due to tracheobronchial remnants (40). However, the definitive diagnosis is always histological.

The appropriate choice of treatment depends on the location, severity, and type of stenosis. CES caused by TBR tends to require surgical correction, whereas CES caused by FMS is usually treated only by bougienage or dilatation. The EM type is typically managed by endoscopic dilation or excision (39, 41).

Acquired esophageal stenosis (AES) include stenosis secondary to esophagitis (peptic, eosinophilic, pill-related), former esophageal surgery or esophageal burns due to caustic ingestion or radiation therapy (4).

3.3 Esophageal rings

A Schatzki ring (SR) is a rare finding which consists in a thin, circumferential submucosal ring (1–2 mm in thickness) that protrudes into the lumen of the distal esophagus at the gastroesophageal junction (42). Studies have shown an association with EoE (43) and peptic esophagitis (44). The incidence in children is not well established. One study demonstrated that a SR was present in 0.2% of pediatric patients undergoing an EGD or an upper GI series (45). Patients with a SR usually presents with dysphagia. Other symptoms are GERD, vomiting, food bolus, choking, chest pain and abdominal pain (42, 45).

3.4 Esophageal diverticula

Congenital diverticula are secondary to increased luminal pressure due to uncoordinated swallowing, impaired relaxation, and muscular spasm, that lead to in-utero formation of an esophageal diverticulum. Acquired diverticula are pulsion diverticula that develop in children with esophageal dysmotility disorders, functional or mechanical obstruction, weakness of the esophageal wall or previous surgery. Only few cases are reported in Literature.

Patients can present with dysphagia, choking, respiratory distress or vomiting of undigested food. The diagnostic gold standard is barium esophagogram (46, 47).

3.5 Esophageal duplication cyst

Esophageal duplication cyst (EDC) is a rare congenital malformation characterized by the presence of a cystic mass that may or not, communicate with the esophageal lumen. It is usually located in the distal esophagus. A high-volume pediatric center cited 68 cases in a period of observation of 58 years (48). EDC is mostly an incidental finding, but it seldom presents with persistent stridor, dyspnea and dysphagia during childhood. EGD and thoracic CT and MRI can be useful to identify the cyst. Histopathological confirmation is needed to confirm diagnosis (49).

4. Extrinsic compression

4.1 Congenital vascular anomalies

The presence of malformations of the aortic arch have been associated to dysphagia due to the extrinsic compression of the esophageal lumen. Dysphagia lusoria (DL), determined by the presence of an aberrant right subclavian artery *("arteria lusoria"*), is the most common abnormality with a prevalence ranging between 0.5% and 1.8%. Other vascular anomalies causing esophageal dysphagia include a persistent right aortic arch with aberrant left subclavian artery, a tortuous or aneurysmal thoracic aorta (*"dysphagia aortica"*), double aortic arch and left atrial enlargement (50). Down's Syndrome and congenital heart disease have been associated to an increased prevalence of aortic arch vascular abnormalities.

Other than dysphagia, children with congenital vascular anomalies often experience respiratory symptoms secondary to tracheal compression.

Barium X-ray series followed by either a angio-CT or MRI scan are used to confirm diagnosis (51).

QUESTIONNAIRES AND CLINICAL SCALES

Supplementary table 2. Eating Assessment Tool (EAT-10) [52]

TO WHAT EXTENT ARE THE FOLLOWING SCENARIOS PROBLEMATIC FOR YOU?
(0 = no problem, 4 = severe problem)

**1.**	My swallowing problem caused me to lose weight	0	1	2	3	4
**2.**	My swallowing problem interferes with my ability to go out for meals	0	1	2	3	4
**3.**	Swallowing liquids takes extra effort	0	1	2	3	4
**4.**	Swallowing solids takes extra effort	0	1	2	3	4
**5.**	Swallowing pills takes extra effort	0	1	2	3	4
**6.**	Swallowing is painful	0	1	2	3	4
**7.**	The pleasure of eating is affected by my swallowing	0	1	2	3	4
**8.**	When I swallow food sticks in my throat	0	1	2	3	4
**9.**	I cough when I eat	0	1	2	3	4
**10.**	Swallowing is stressful	0	1	2	3	4
**TOTAL SCORE** (a score $\geq$ 3 is abnormal)						

Supplementary table 3. The Pediatric Version of the Eating Assessment Tool (PEDI-EAT-10) [53]

The Pediatric Version of the Eating Assessment Tool helps us to determine if your child has a swallowing difficulty. Answer each question by thinking back over a typical one-month period and select the number that best described your child. 0 means no problem, 4 means severe problem. Every item defines a problem, and this does not mean that every child has the mentioned problem. If your child does not have the mentioned problem, please choose "0" which means that <no problem> in other words <my child does not have this problem>. If your child encounters that problem, please rate the severity of that problem between 1 and 4 for your child. Thank you.

Answer each question by writing the number of points in the boxes
(0 = no problem, 4 = severe problem)

**1.**	My child does not gain weight due to his/her swallowing problem	0	1	2	3	4
**2.**	Swallowing problem of my child interferes with our ability to go out for meals	0	1	2	3	4
**3.**	Swallowing liquids takes extra effort for my child	0	1	2	3	4
**4.**	Swallowing solids takes extra effort for my child	0	1	2	3	4
**5.**	My child gags during swallowing	0	1	2	3	4
**6.**	My child acts like he/she is in pain while swallowing	0	1	2	3	4
**7.**	My child does not want to eat	0	1	2	3	4
**8.**	Food sticks in my child's throat and my child chokes while eating	0	1	2	3	4
**9.**	My child coughs while eating	0	1	2	3	4
**10.**	Swallowing is stressful for my child	0	1	2	3	4
**TOTAL SCORE** (a score $\geq$ 4 is abnormal and has a sensitivity of 91.3% and specificity of 98.8% to predict penetration/aspiration)						

Supplementary table 4. Reflux Symptom Index [54]

Within the last month, how did the following problems affect you?
0 = no problem, 5 = severe problem

**1.**	Hoarseness or a problem with your voice	0	1	2	3	4	5
**2.**	Clearing your throat	0	1	2	3	4	5
**3.**	Excess throat mucus or postnatal drip	0	1	2	3	4	5
**4.**	Difficulty swallowing food, liquids, or pills	0	1	2	3	4	5
**5.**	Coughing after you ate or after lying down	0	1	2	3	4	5
**6.**	Breathing difficulties or choking episodes	0	1	2	3	4	5
**7.**	Troublesome or annoying cough	0	1	2	3	4	5
**8.**	Sensations of something sticking in your throat or a lump in your throat	0	1	2	3	4	5
**9.**	Heartburn, chest pain, indigestion, or stomach acid coming up	0	1	2	3	4	5
**TOTAL SCORE** (RSI is considered indicative of significant reflux disease when > 13, indeterminate between 10 and 13, and normal if < 10)							

Supplementary table 5. Pediatric Eosinophilic Esophagitis Symptom Score (PEESS version 2.0; Children and teens report (ages 8-18) [55]

Tell us about your problems with EoE in the past month
Frequency: 0 = never, 1 = almost never (less than once a week), 2= sometimes (1 or more times a week), 3 = often (1 time a day), almost always (2 or more times a day) 4 = severe problem)
Severity: 0 = not bad at all, 1 = a little bad, 2= kind of bad, 3 = bad, 4 = very bad

**1.**	How often do you have chest pain, ache, or hurt?	0	1	2	3	4
**2.**	How bad is the chest pain, ache, or hurt?	0	1	2	3	4
**3.**	How often do you have heartburn (burning in your chest, mouth, or throat)?	0	1	2	3	4
**4.**	How bad is your heartburn (burning in your chest, mouth, or throat)?	0	1	2	3	4
**5.**	How often do you have stomach aches or belly aches?	0	1	2	3	4
**6.**	How bad are the stomach aches or belly aches?	0	1	2	3	4
**7.**	How often do you have trouble swallowing?	0	1	2	3	4
**8.**	How bad is the trouble swallowing?	0	1	2	3	4
**9.**	How often do you feel like food gets stuck in your throat or chest?	0	1	2	3	4
**10.**	How bad is it when food gets stuck in your throat or chest?	0	1	2	3	4
**11.**	How often do you need to drink a lot to help swallow your food?	0	1	2	3	4
**12.**	How bad is it if you don't drink a lot to help swallow your food?	0	1	2	3	4
**13.**	How often do you vomit (throw up)?	0	1	2	3	4
**14.**	How bad is the vomiting (throwing up)?	0	1	2	3	4
**15.**	How often do you feel nauseous (feel like you're going to throw up, but don't)?	0	1	2	3	4

**16.**	How bad is the nausea (feeling like you're going to throw up, but don't)?	0	1	2	3	4
**17.**	How often does food come back up your throat when eating?	0	1	2	3	4
**18.**	How bad is the food coming back up your throat when eating?	0	1	2	3	4
**19.**	How often do you eat less food than others?	0	1	2	3	4
**20.**	How often do you need more time to eat than others?	0	1	2	3	4
**TOTAL SCORE 0-80**<br>Higher scores are indicative of more frequent and/or severe symptoms						

Supplementary table 6. Pediatric Eosinophilic Esophagitis Symptom Score (PEESS version 2.0; Parent Report for Children and Teens (ages 2-18) [55]

Tell us about your child's problems with EoE in the past month
Frequency: 0 = never, 1 = almost never (less than once a week), 2= sometimes (1 or more times a week), 3 = often (1 time a day), almost always (2 or more times a day) 4 = severe problem)
Severity: 0 = not bad at all, 1 = a little bad, 2= kind of bad, 3 = bad, 4 = very bad

**1.**	How often does your child have chest pain, ache, or hurt?	0	1	2	3	4
**2.**	How bad is your child's chest pain, ache, or hurt?	0	1	2	3	4
**3.**	How often does your child have heartburn (burning in your chest, mouth, or throat)?	0	1	2	3	4
**4.**	How bad is your child's heartburn (burning in your chest, mouth, or throat)?	0	1	2	3	4
**5.**	How often does your child have stomach aches or belly aches?	0	1	2	3	4
**6.**	How bad are your child's stomach aches or belly aches?	0	1	2	3	4
**7.**	How often does your child have trouble swallowing?	0	1	2	3	4
**8.**	How bad is your child's trouble swallowing?	0	1	2	3	4
**9.**	How often does your child feel like food gets stuck in his/her throat or chest?	0	1	2	3	4
**10.**	How bad is it when your child gets food stuck in his/her throat or chest?	0	1	2	3	4
**11.**	How often does your child need to drink a lot to help swallow food?	0	1	2	3	4
**12.**	How bad is it when your child needs to drink a lot to help swallow food?	0	1	2	3	4
**13.**	How often does your child vomit (throw up)?	0	1	2	3	4
**14.**	How bad is your child's vomiting (throwing up)?	0	1	2	3	4
**15.**	How often does your child feel nauseous (feel like throwing up, but doesn't)?	0	1	2	3	4
**16.**	How bad is your child's nausea (feeling like throwing up, but doesn't)?	0	1	2	3	4
**17.**	How often does your child have food come back up in his/her throat when eating?	0	1	2	3	4
**18.**	How bad is it when food comes back up in your child's throat?	0	1	2	3	4
**19.**	How often does your child eat less food than others?	0	1	2	3	4
**20.**	How often does your child need more time to eat than others?	0	1	2	3	4
**TOTAL SCORE 0-80**<br>Higher scores are indicative of more frequent and/or severe symptoms						

Supplementary table 7. Eckardt score for esophageal achalasia [56, 57]

Symptom	Score			
Dysphagia	None	Occasional	Daily	At every meal
Regurgitation	None	Occasional	Daily	At every meal
Chest pain	None	Occasional	Daily	At every meal
Weight loss (kg)	None	Occasional	Daily	Several times per day
**TOTAL SCORE** The final score is the sum of the four components scores, ranging from 0 to 12. A threshold value of > 3 is considered to be a suboptimal outcome after treatment.				

References

1. Rosen R, Vandenplas Y, Singendonk M, Cabana M, DiLorenzo C, Gottrand F, et al. Pediatric Gastroesophageal Reflux Clinical Practice Guidelines: Joint Recommendations of the North American Society for Pediatric Gastroenterology, Hepatology, and Nutrition and the European Society for Pediatric Gastroenterology, Hepatology, and Nutrition. J Pediatr Gastroenterol Nutr. 2018;66(3):516-54.
2. Martigne L, Delaage PH, Thomas-Delecourt F, Bonnelye G, Barthelemy P, Gottrand F. Prevalence and management of gastroesophageal reflux disease in children and adolescents: a nationwide cross-sectional observational study. Eur J Pediatr. 2012;171(12):1767-73.
3. Poddar U. Gastroesophageal reflux disease (GERD) in children. Paediatr Int Child Health. 2019;39(1):7-12.
4. Ghiselli A, Bizzarri B, Ferrari D, Manzali E, Gaiani F, Fornaroli F, et al. Endoscopic dilation in pediatric esophageal strictures: a literature review. Acta Biomed. 2018;89(8-S):27-32.
5. Arias A, Perez-Martinez I, Tenias JM, Lucendo AJ. Systematic review with meta-analysis: the incidence and prevalence of eosinophilic oesophagitis in children and adults in population-based studies. Aliment Pharmacol Ther. 2016;43(1):3-15.
6. Shaheen NJ, Mukkada V, Eichinger CS, Schofield H, Todorova L, Falk GW. Natural history of eosinophilic esophagitis: a systematic review of epidemiology and disease course. Dis Esophagus. 2018;31(8).
7. Papadopoulou A, Koletzko S, Heuschkel R, Dias JA, Allen KJ, Murch SH, et al. Management guidelines of eosinophilic esophagitis in childhood. J Pediatr Gastroenterol Nutr. 2014;58(1):107-18.
8. Tringali A, Thomson M, Dumonceau JM, Tavares M, Tabbers MM, Furlano R, et al. Pediatric gastrointestinal endoscopy: European Society of Gastrointestinal Endoscopy (ESGE) and European Society for Paediatric Gastroenterology Hepatology and Nutrition (ESPGHAN) Guideline Executive summary. Endoscopy. 2017;49(1):83-91.
9. Dellon ES, Liacouras CA, Molina-Infante J, Furuta GT, Spergel JM, Zevit N, et al. Updated International Consensus Diagnostic Criteria for Eosinophilic Esophagitis: Proceedings of the AGREE Conference. Gastroenterology. 2018;155(4):1022-33 e10.
10. Hoversten P, Kamboj AK, Katzka DA. Infections of the esophagus: an update on risk factors, diagnosis, and management. Dis Esophagus. 2018;31(12).
11. Bordea MA, Pirvan A, Gheban D, Silaghi C, Lupan I, Samasca G, et al. Infectious Esophagitis in Romanian Children: From Etiology and Risk Factors to Clinical Characteristics and Endoscopic Features. J Clin Med. 2020;9(4).
12. Patel NC, Caicedo RA. Esophageal infections: an update. Curr Opin Pediatr. 2015;27(5):642-8.
13. Ramaswamy K, Jacobson K, Jevon G, Israel D. Esophageal Crohn disease in children: a clinical spectrum. J Pediatr Gastroenterol Nutr. 2003;36(4):454-8.
14. Ammoury RF, Pfefferkorn MD. Significance of esophageal Crohn disease in children. J Pediatr Gastroenterol Nutr. 2011;52(3):291-4.
15. Rahhal RM, Banerjee S, Jensen CS. Pediatric Crohn disease presenting as an esophageal stricture. J Pediatr Gastroenterol Nutr. 2007;45(1):125-9.
16. Bordea MA, Pirvan A, Sarban C, Margescu C, Leucuta D, Samasca G, et al. Pill -Induced Erosive Esophagitis in Children. Clujul Med. 2014;87(1):15-8.
17. Hu SW, Chen AC, Wu SF. Drug-Induced Esophageal Ulcer in Adolescent Population: Experience at a Single Medical Center in Central Taiwan. Medicina (Kaunas). 2021;57(12).
18. Nesheiwat Z, Akbar H, Kahloon A, Mahajan K. Radiation Esophagitis. StatPearls. Treasure Island (FL)2022.
19. Yadlapati R, Kahrilas PJ, Fox MR, Bredenoord AJ, Prakash Gyawali C, Roman S, et al. Esophageal motility disorders on high-resolution manometry: Chicago classification version 4.0((c)). Neurogastroenterol Motil. 2021;33(1):e14058.
20. Vaezi MF, Pandolfino JE, Yadlapati RH, Greer KB, Kavitt RT. ACG Clinical Guidelines: Diagnosis and Management of Achalasia. Am J Gastroenterol. 2020;115(9):1393-411.
21. Marlais M, Fishman JR, Fell JM, Haddad MJ, Rawat DJ. UK incidence of achalasia: an 11-year national epidemiological study. Arch Dis Child. 2011;96(2):192-4.
22. Farrokhi F, Vaezi MF. Idiopathic (primary) achalasia. Orphanet J Rare Dis. 2007;2:38.

23. Islam S. Achalasia. Semin Pediatr Surg. 2017;26(2):116-20.
24. Hallal C, Kieling CO, Nunes DL, Ferreira CT, Peterson G, Barros SG, et al. Diagnosis, misdiagnosis, and associated diseases of achalasia in children and adolescents: a twelve-year single center experience. Pediatr Surg Int. 2012;28(12):1211-7.
25. Tashiro J, Petrosyan M, Kane TD. Current management of pediatric achalasia. Transl Gastroenterol Hepatol. 2021;6:33.
26. Richter JE. Chicago Classification Version 4.0 and Its Impact on Current Clinical Practice. Gastroenterol Hepatol (N Y). 2021;17(10):468-75.
27. Li SC. Scleroderma in Children and Adolescents: Localized Scleroderma and Systemic Sclerosis. Pediatr Clin North Am. 2018;65(4):757-81.
28. Torok KS. Pediatric scleroderma: systemic or localized forms. Pediatr Clin North Am. 2012;59(2):381-405.
29. Martini G, Foeldvari I, Russo R, Cuttica R, Eberhard A, Ravelli A, et al. Systemic sclerosis in childhood: clinical and immunologic features of 153 patients in an international database. Arthritis Rheum. 2006;54(12):3971-8.
30. Kadakuntla A, Juneja A, Sattler S, Agarwal A, Panse D, Zakhary N, et al. Dysphagia, reflux and related sequelae due to altered physiology in scleroderma. World J Gastroenterol. 2021;27(31):5201-18.
31. Nassar N, Leoncini E, Amar E, Arteaga-Vazquez J, Bakker MK, Bower C, et al. Prevalence of esophageal atresia among 18 international birth defects surveillance programs. Birth Defects Res A Clin Mol Teratol. 2012;94(11):893-9.
32. Dingemann C, Eaton S, Aksnes G, Bagolan P, Cross KM, De Coppi P, et al. ERNICA Consensus Conference on the Management of Patients with Esophageal Atresia and Tracheoesophageal Fistula: Diagnostics, Preoperative, Operative, and Postoperative Management. Eur J Pediatr Surg. 2020;30(4):326-36.
33. Rayyan M, Allegaert K, Omari T, Rommel N. Dysphagia in Children with Esophageal Atresia: Current Diagnostic Options. Eur J Pediatr Surg. 2015;25(4):326-32.
34. Serrao E, Santos A, Gaivao A, Tavares A, Ferreira S. Congenital esophageal stenosis: a rare case of dysphagia. J Radiol Case Rep. 2010;4(6):8-14.
35. Amae S, Nio M, Kamiyama T, Ishii T, Yoshida S, Hayashi Y, et al. Clinical characteristics and management of congenital esophageal stenosis: a report on 14 cases. J Pediatr Surg. 2003;38(4):565-70.
36. Ramesh JC, Ramanujam TM, Jayaram G. Congenital esophageal stenosis: report of three cases, literature review, and a proposed classification. Pediatr Surg Int. 2001;17(2-3):188-92.
37. Pokieser P, Schima W, Schober E, Bohm P, Stacher G, Levine MS. Congenital esophageal stenosis in a 21-year-old man: clinical and radiographic findings. AJR Am J Roentgenol. 1998;170(1):147-8.
38. Trappey AF, 3rd, Hirose S. Esophageal duplication and congenital esophageal stenosis. Semin Pediatr Surg. 2017;26(2):78-86.
39. Usui N, Kamata S, Kawahara H, Sawai T, Nakajima K, Soh H, et al. Usefulness of endoscopic ultrasonography in the diagnosis of congenital esophageal stenosis. J Pediatr Surg. 2002;37(12):1744-6.
40. Kawahara H, Oue T, Okuyama H, Kubota A, Okada A. Esophageal motor function in congenital esophageal stenosis. J Pediatr Surg. 2003;38(12):1716-9.
41. Jones DW, Kunisaki SM, Teitelbaum DH, Spigland NA, Coran AG. Congenital esophageal stenosis: the differential diagnosis and management. Pediatr Surg Int. 2010;26(5):547-51.
42. Buckley K, Buonomo C, Husain K, Nurko S. Schatzki ring in children and young adults: clinical and radiologic findings. Pediatr Radiol. 1998;28(11):884-6.
43. Nurko S, Teitelbaum JE, Husain K, Buonomo C, Fox VL, Antonioli D, et al. Association of Schatzki ring with eosinophilic esophagitis in children. J Pediatr Gastroenterol Nutr. 2004;38(4):436-41.
44. Scharschmidt BF, Watts HD. The lower esophageal ring and esophageal reflux. Am J Gastroenterol. 1978;69(5):544-9.
45. Towbin AJ, Diniz LO. Schatzki ring in pediatric and young adult patients. Pediatr Radiol. 2012;42(12):1437-40.
46. Smith CD. Esophageal strictures and diverticula. Surg Clin North Am. 2015;95(3):669-81.
47. Lindholm EB, Hansbourgh F, Upp JR, Jr., Cilloniz R, Lopoo J. Congenital esophageal diverticulum - a case report and review of literature. J Pediatr Surg. 2013;48(3):665-8.

48. Nobuhara KK, Gorski YC, La Quaglia MP, Shamberger RC. Bronchogenic cysts and esophageal duplications: common origins and treatment. J Pediatr Surg. 1997;32(10):1408-13.
49. Sun CF, Chen CH, Ke PZ, Ho TL, Lin CH. Esophageal duplication cyst presenting with stridor in a child with congenital pulmonary airway malformation: A case report and literature review. Medicine (Baltimore). 2019;98(28):e16364.
50. Hryhorczuk AL, Lee EY, Eisenberg RL. Esophageal abnormalities in pediatric patients. AJR Am J Roentgenol. 2013;201(4):W519-32.
51. Levitt B, Richter JE. Dysphagia lusoria: a comprehensive review. Dis Esophagus. 2007;20(6):455-60.
52. Belafsky PC, Mouadeb DA, Rees CJ, Pryor JC, Postma GN, Allen J, et al. Validity and reliability of the Eating Assessment Tool (EAT-10). Ann Otol Rhinol Laryngol. 2008;117(12):919-24.
53. Soyer T, Yalcin S, Arslan SS, Demir N, Tanyel FC. Pediatric Eating Assessment Tool-10 as an indicator to predict aspiration in children with esophageal atresia. J Pediatr Surg. 2017;52(10):1576-9.
54. Belafsky PC, Postma GN, Koufman JA. Validity and reliability of the reflux symptom index (RSI). J Voice. 2002;16(2):274-7.
55. Franciosi JP, Hommel KA, DeBrosse CW, Greenberg AB, Greenler AJ, Abonia JP, et al. Development of a validated patient-reported symptom metric for pediatric eosinophilic esophagitis: qualitative methods. BMC Gastroenterol. 2011;11:126.
56. Taft TH, Carlson DA, Triggs J, Craft J, Starkey K, Yadlapati R, et al. Evaluating the reliability and construct validity of the Eckardt symptom score as a measure of achalasia severity. Neurogastroenterol Motil. 2018;30(6):e13287.
57. Eckardt VF, Aignherr C, Bernhard G. Predictors of outcome in patients with achalasia treated by pneumatic dilation. Gastroenterology. 1992;103(6):1732-8.
